# “Irregularities” in the Texas Quarantine Department

**Published:** 1902-01

**Authors:** 


"Irregularities" in the Texas Quarantine Department.—A first-class sensation was sprung in Austin about January 1st, inst., when Dr. Geo. R. Tabor, the newly appointed State Health salary had been drawn on W. L. Moody's bank at Galveston on the 1st on account of ill health it is said) discovered that a warrant for $300 in his favor for services as special inspector at New Orleans prior to his succession to Dr. Blunt had been paid, he getting the money, and that a check for the same amount and for the same salary had been drawn on W. S. Moody's bank at Galveston on the "fumigating fund" on deposit there—he *not* getting the money. This led to an examination of the books of the department, and similar "irregularities" were unearthed—the exact amount of the "irregularities" not having at this writing, January 2, been ascertained. Dr. Blunt, the retiring State Health Officer, entrusted the management of that part of the work, involving approval of bills and seeing to the issue of warrants therefor, and the keeping of the account of the fumigating fund to Dr. I. J. Jones, his secretary, and Jones signed the State Health Officer's name to such vouchers by his authority, it is said; hence, Jones is the "responsible party." Meantime, the Legislature last September, having abolished the office of secretary, turned Jones out. Dr. Blunt resigned about November 1st, on account of ill health, and Dr. Tabor took charge. Dr. Jones had business in Mexico, where he is at this writing. Just what the outcome will be we will have to wait and see. Dr. Blunt's bond for $10,000 is as good as gold. Nothing can be made out of Jones. The plot thickens.

Dr. Tabor also discovered "irregularities" in the fumigating fund at Sabine Pass and promptly removed Dr. L. A. Tackaberry, management of that part of the work involving approval of bills the quarantine inspector at that point. A warrant was issued for his arrest by order of the State Health Officer, but he skipped.

